# Coexpression Network Analysis of miRNA-142 Overexpression in Neuronal Cells

**DOI:** 10.1155/2015/921517

**Published:** 2015-10-11

**Authors:** Ishwor Thapa, Howard S. Fox, Dhundy Bastola

**Affiliations:** ^1^College of Information Science and Technology, University of Nebraska Omaha, Omaha, NE 68182, USA; ^2^Department of Pharmacology and Experimental Neuroscience, University of Nebraska Medical Center, Omaha, NE 68198, USA

## Abstract

MicroRNAs are small noncoding RNA molecules, which are differentially expressed in diverse biological processes and are also involved in the regulation of multiple genes. A number of sites in the 3′ untranslated regions (UTRs) of different mRNAs allow complimentary binding for a microRNA, leading to their posttranscriptional regulation. The miRNA-142 is one of the microRNAs overexpressed in neurons that is found to regulate *SIRT1* and *MAOA* genes. Differential analysis of gene expression data, which is focused on identifying up- or downregulated genes, ignores many relationships between genes affected by miRNA-142 overexpression in a cell. Thus, we applied a correlation network model to identify the coexpressed genes and to study the impact of miRNA-142 overexpression on this network. Combining multiple sources of knowledge is useful to infer meaningful relationships in systems biology. We applied coexpression model on the data obtained from wild type and miR-142 overexpression neuronal cells and integrated miRNA seed sequence mapping information to identify genes greatly affected by this overexpression. Larger differences in the enriched networks revealed that the nervous system development related genes such as *TEAD2, PLEKHA6*, and *POGLUT1* were greatly impacted due to miRNA-142 overexpression.

## 1. Introduction

MicroRNAs are small noncoding RNA molecules known to regulate gene expression at the transcriptional and posttranscriptional levels in a cell. The abnormal levels of microRNAs (miRNAs) may alter target gene expression or protein expression leading to pathogenesis of certain diseases. A large number of diseases including various forms of cancers have been implicated by aberrant expression of different miRNAs [1, 2]. Several studies have also explored miRNA and disease associations [3, 4]. In recent years, many* in vitro* gene expression profiling studies on overexpression or inhibition of miRNAs have been completed to discover novel miRNAs and their targets leading to innovative findings of miRNA associated diseases.

Differential gene expression studies focus on identifying genes that are up- and downregulated in a given experimental condition. However, it fails to capture the dependencies between genes and their relationships to other coexpressed genes. For this reason, network has become an increasingly popular model that analyzes relationships between genes/proteins within a biological system. Correlation networks based on coexpression have been extensively used [5]. Previously, a number of network properties have been associated with various biological processes, some of which include gene regulation, protein complex, and core diseasome within a given network [6–10]. Additionally, multiple networks have been compared using graph alignment algorithms to examine substructures that were conserved or destroyed between networks [11, 12].

A survey of the literature shows that miRNA-142 is upregulated in neurons and its overexpression through stable gene transfer downregulates the expression of NAD-dependent deacetylase Sirtuin 1 (*SIRT1*) gene and consequently the expression of the monoamine oxidase (*MAOA*) gene [13]. Such indirect regulations are inherent properties of a biological system and are difficult to capture in a gene expression study using differential analysis. Since miRNAs regulate target gene expression levels in a cell through complementary binding of its sequence to the 3′ untranslated region (UTR) of the mRNA it targets, one may use miRNA seed sequence mapping information to explain the downregulation of targeted genes. However, this gene regulation is not consistent in all of the miRNA seed sequence targets and conversely, genes that are regulated do not always have the seed sequence mapped. Therefore, in the present study, we propose a coexpression based method which incorporates multiple knowledge sources to find relevant target genes.

The correlation networks are built with the gene expression profiling data from (1) control (called the miR-null network) and (2) neuronal cells overexpressing miRNA-142 (called the miR-142 network) and are enriched with different knowledge sources derived from miRNA-mRNA seed mapping and differential gene expression. The comparison between the two enriched networks resulted in a number of genes, which were greatly affected by the overexpression of miRNA-142. Analyzing only the differential expression and miRNA-mRNA seed mapping led to noisy results. However, when coexpression networks were enriched with the different knowledge sources mentioned above, the networks highlighted key genes impacted by the biological transformation. The results showed some genes common between the coexpression network enriched with seed sequence mapping and the differentially expressed (DE) genes. These observations along with the approach taken in the present study are expected to be valuable in obtaining a comprehensive list of genes, which might be directly or indirectly regulated by the overexpression of miRNA in the cells.

## 2. Materials and Methods

A pipeline using the existing and new tools was developed in the process of this study. The Affymetrix power tool (APT) software package was used to retrieve exon level expression data and the 3′ UTR mapping was performed using Perl programming. Other analyses were accomplished using free and open source (FOSS) tools like R, Cytoscape, Limma, and GOFunction [14–18].

### 2.1. Dataset

Affymetrix gene expression data of RNA extracted from three independent overexpressed miRNA-142 clones and three stable clones with no miRNA overexpression were processed with the APT package for background correction, normalization, and summarizing probe sets. This data is available in NCBI GEO identified as GSE50133 series. The default parameters were used with* apt-probeset-summarize* and quantile normalization was applied in* rma-sketch* to obtain gene expression values for all samples. This gene level expression data contained three columns of miR-null replicates and three columns of miR-142 replicates. In order to find complementary mapping of miRNA seed sequence to 3′ UTR of mRNA, miRNA-142 seed sequence information was downloaded from TargetScan website and all the human mRNA sequences were obtained from Ensembl FTP site [19, 20].

### 2.2. Network Construction

Pearson correlation coefficients were calculated for all pairs of genes in miR-142 overexpressed dataset and miR-null dataset. To address multiple testing problem while computing the correlation coefficients for all pairs of genes, various *P* value adjustment methods such as “Holm,” “Hochberg,” and “Bonferroni” were applied. All the adjusted methods produced the same result and the minimum correlation coefficient was found to be 0.9998. Using the sample size estimation software (http://www.cct.cuhk.edu.hk/stat/other/correlation.htm) based on [21], it was derived that, with significance level at 0.01 and power of 80%, the number of samples could be of size three, if the minimum correlation was set to 0.9998. Thus, with confidence, the correlation coefficient values were represented as edges and genes as nodes to construct two independent networks. Only positive correlations were considered to model the coexpression as a positive effect. This resulted in two small, undirected networks, named here as miR-142 and miR-null. The Cytoscape tool was used to visualize the networks and enrich them with various information such as differential expression and miRNA seed mapping.

### 2.3. miRNA-mRNA Seed Matching

**Figure 1:**
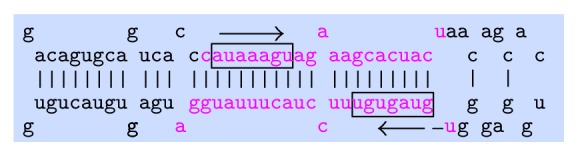
Stem-loop structure of miRNA-142 from miRBase website [23]. The highlighted boxes represent seed sequences for miR-142-5p and miR-142-3p.

The miRNA-142 is one of the several miRNAs, which has both 5′ and 3′ arms of the stem-loop that can independently participate in regulation [22].  Figure 1 shows the miRNA-142 stem-loop structure obtained from the miRBase [23]. For simplicity, 5′ arm of miRNA-142 is written as miR-142-5p and 3′ arm as miR-142-3p.

The miRNA-mRNA complementary binding, which may result in the mRNA degradation or translation repression, is not always exact. Studies have shown that the binding may include an imperfect site [24]. Therefore, the exact seed sequences of miR-142-5p (AUAAAGU), miR-142-3p (GUAGUGU), and their 1-base mutated seed sequences were complementarily mapped to 3′ UTR of all the transcripts obtained from human genome. The human mRNA sequences were downloaded from the Ensembl FTP site [19, 20]. The list of both the exact and inexact seed sequences is presented in Table 1.

### 2.4. Differential Analysis

Due to small number of samples (three) in each group, we used the “lmFit” and “topTable” procedures from “Limma” package [17]. The genes which were differentially expressed with adjusted *P* values ≤ 0.1 were used in this study. To address multiple testing problem, *P* values were adjusted using “Benjamini and Hochberg” method. Additionally, we have also used the Mann-Whitney (also called the Wilcoxon Rank Sum) test to perform the *t*-test.

### 2.5. Functional Analysis

The functional analysis program was written in R using GOFunction package [18]. The GOFunction tool is an enrichment analysis tool for Gene Ontology (GO). One advantage of using this tool is that it combines redundant GO terms based on the GO structure and gives statistically interpretable enriched GO terms. By default, the *P* value adjustment method selected was the “BY” option representing the “Benjamini, Hochberg, and Yekutieli” method.  Table 5 shows GO terms enriched in the differentially expressed gene list obtained from the GOFunction.

## 3. Results

The goal of this study was to understand the changes in a cell due to the overexpression of miRNA-142 and its downstream effect on cellular functions.

In our results, we first show why the miRNA seed mapping and differential expression analysis could lead to noisy results (with many false positives and true negatives). Next, we show the impact of miR-142 overexpression on the coexpression networks of genes. These networks are further enriched with miRNA-mRNA seed sequence mapping, which illustrates that the networks are biologically relevant. We apply differential analysis result to our network and find an overlap between the differentially expressed genes and the nodes (genes) in the network. Functional analysis of the differentially expressed genes reveals enrichment in nervous system/neuron related genes.

### 3.1. Overlap between DE Genes and Seed Sequence Mapping

We independently identified genes which are differentially expressed and also mapped seed sequence of miR-142-5p and miR-142-3p to all the 3-prime untranslated regions (3p UTRs) of human mRNAs. We discuss this process in Section 2. Here, we show Table 2 that contains number of genes that are up- and downregulated and also have miRNA seed sequence mapping to their 3p UTRs. We observed that both the upregulated and the downregulated genes have large fraction (74%) of genes with matching seed sequence.

Since the miRNA-mRNA complementary binding may include an imperfect site, we also compared the inexact seed sequence mapping results with the differentially up- and downregulated gene lists (discussed more in Section 2). With inexact mapping, we also observed similar results as seen for exact seed sequence mapping.

**Figure 2:**
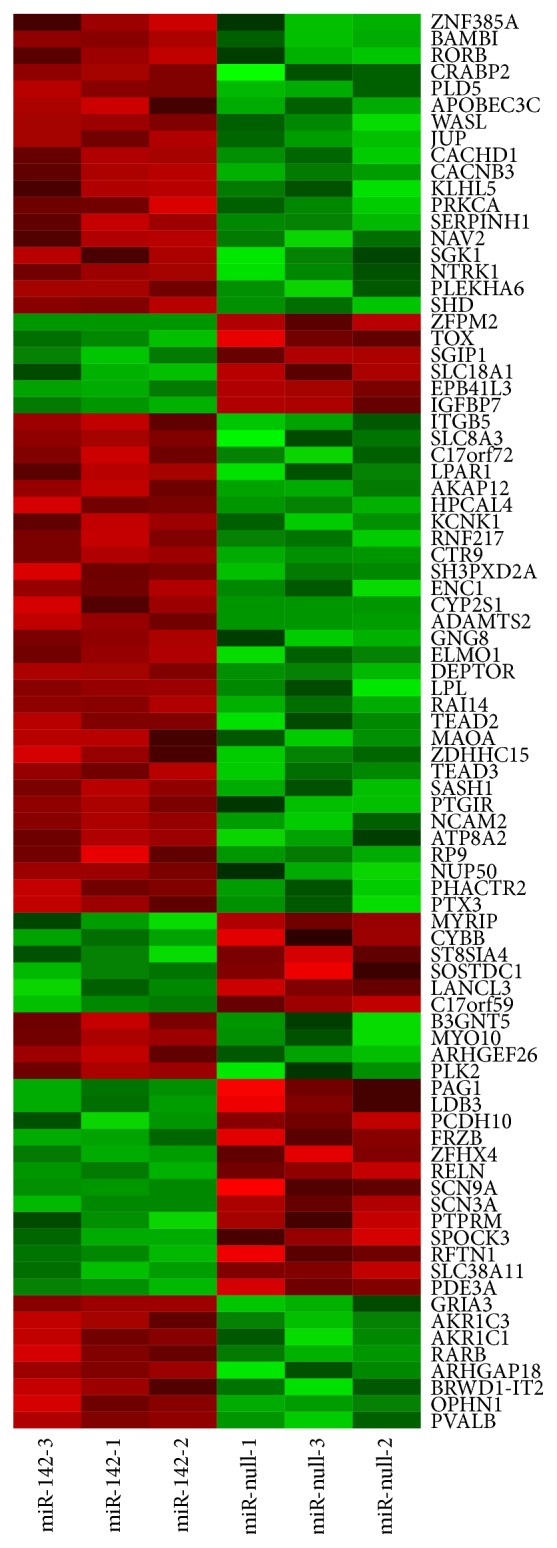
Heatmap showing normalized gene expression values for 85 DE genes obtained from “Limma” package.

When we analyzed the differential expression using the “Limma” package (see Figure 2), we found 85 genes were differentially expressed with *P* values ≤ 0.1 (*P* values corrected by Benjamini and Hochberg method). We checked these 85 genes with the DE list obtained from the “Wilcoxon Rank Sum (WRS)” test and found all of them included. Out of 85 DE genes obtained using “Limma,” 63 (20 upregulated and 43 downregulated) genes had miRNA-142 seed sequence mapping. We observed that both up- and downregulated genes have seed sequence mapping and, conversely, not all the differentially expressed genes have seed sequence mapping. The 17 downregulated genes and 5 upregulated genes (*P* value ≤ 0.1) did not have any miR-142 seed mapping. Hence, direct relationship between gene regulation and a microRNA cannot be inferred only by looking into seed sequence mapping. Moreover, the result in Table 2 suggests that the miRNA-mRNA seed sequence mapping may directly or indirectly impact gene regulation. The Chi-square test was inconclusive to suggest any relation between up-/downregulation and preference to either of the arms of miRNA-142 (*χ* ^2^ = 2.49, df = 2, and *P* value = 0.2878).

### 3.2. Distinct Coexpressed Networks in miRNA-Null and miRNA-142 Overexpression

**Figure 3:**
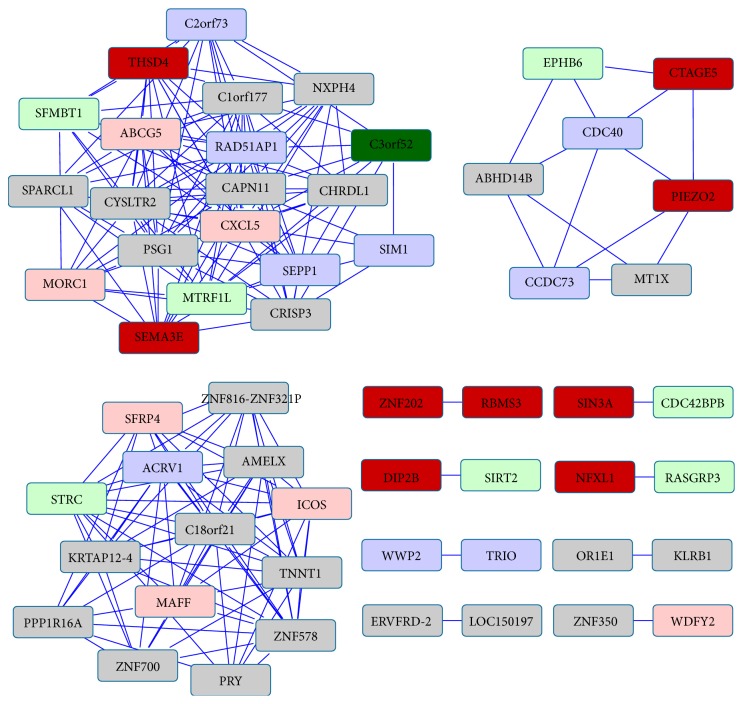
The miR-null coexpression network enriched with miRNA-mRNA seed sequence mapping. Node represents gene name and an edge between two nodes (genes) represents coexpression of the genes. Color of the node represents the miRNA seed sequence mapping onto the 3p UTR of a gene (mRNA). The “dark red” node represents exact miR-142-5p seed mapping, while the “pink” node represents inexact miR-142-5p seed mapping. Similarly, “dark green” node represents exact mapping of miR-142-3p seed and the “light green” node represents inexact mapping. The “blue” node represents genes with inexact mapping for both miR-142-3p and miR-142-5p seed sequences. No node was found to have exact mapping of both miR-142-5p and miR-142-3p.

**Figure 4:**
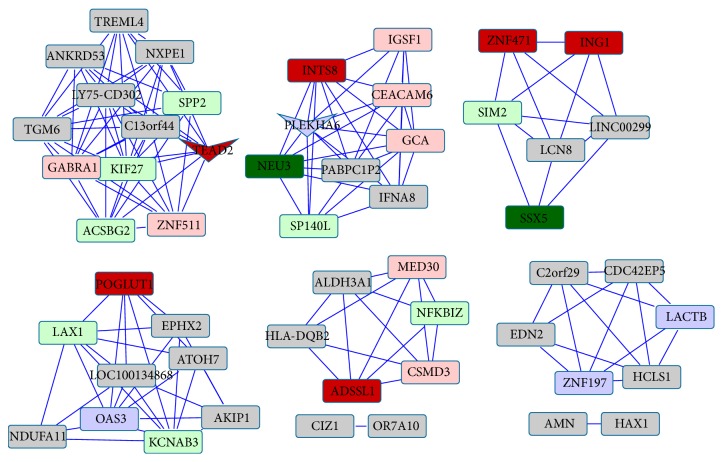
The miR-142 coexpression network enriched with miRNA-mRNA seed sequence mapping and differential expression. Node represents gene name and an edge between two nodes (genes) represents coexpression of the genes. Color of the node represents the miRNA seed sequence mapping onto the 3p UTR of a gene (mRNA). The “dark red” node represents exact miR-142-5p seed mapping, while the “pink” node represents inexact miR-142-5p seed mapping. Similarly, “dark green” node represents exact mapping of miR-142-3p seed and the “light green” node represents inexact mapping. The “blue” node represents genes with inexact mapping for both miR-142-3p and miR-142-5p seed sequences. No node was found with exact mapping of both miR-142-5p and miR-142-3p. The shape of a node represents the differential expression. Down-pointing arrow shaped node represents downregulation in expression.

Coexpressed networks were generated by assigning an edge for Pearson's correlation coefficient between every pair of genes. For addressing false positives and true negatives in large number of multiple testing, we applied different *P* value adjustment methods and obtained the same results. There were a fewer number of edges, which passed through the *P* value adjustment methods. Hence, the coexpressed networks were small-sized networks. No overlap was found between miR-null and miR-142 networks, meaning all the nodes and edges were different. This suggests that there is a big impact in the coexpression of genes due to the miRNA-142 overexpression.  Table 3 shows the size of these networks. All 57 genes in Figure 3 and all 52 genes in Figure 4 are candidate genes of interest because they appear and disappear in these networks due to the miRNA-142 overexpression.

Next, we demonstrate that these networks are also biologically relevant by enriching it with miRNA-mRNA seed sequence mapping. We observe that more than 50% of the nodes (genes) in these networks have miRNA-142 seed sequence mapped to its 3′ UTR.

Table 4 shows the exact number of nodes in these networks with the seed sequence mapping and also in all the genes from human genome. If we take into account the larger probability of inexact seed mapping (21 times more than that of exact mapping because of the 21 different mutated seed sequences shown in Table 1), a large proportion of nodes in the networks are expected to have inexact mapping. However, we observed 18 and 42 nodes with exact and inexact mapping, respectively, which is less than the anticipated ratio. Moreover, if we compare the number of nodes having exact mapping of miR-142-5p and that with again exact mapping of miR-142-3p in each network, we observed higher number of nodes with the exact mapping of miR-142-5p. This suggests that the 5′ arm of miRNA-142 has greater impact than the 3′ arm during the miRNA-142 overexpression. The Chi-square test to check if the seed mapping (exact/inexact) in coexpression networks is independent of matching by different miRNA arms suggested associations between the number of exact/inexact seed mapping targets in coexpression networks and the matching by different arms (*χ* ^2^ = 13.6125, df = 2, and *P* value = 0.0011).

### 3.3. Overlap between DE Genes, Seed Mapping, and the Enriched Network

The enriched network in Figure 4 shows the overlap between the miR-142 coexpressed genes, miRNA-mRNA seed mapping genes, and the differentially expressed genes. There are two genes (*TEAD2* and* PLEKHA6*) which appear in miR-142 coexpression network, have seed mapping, and are downregulated in the miR-142 overexpression. We examine the roles of these two genes in Section 4.

**Figure 5:**
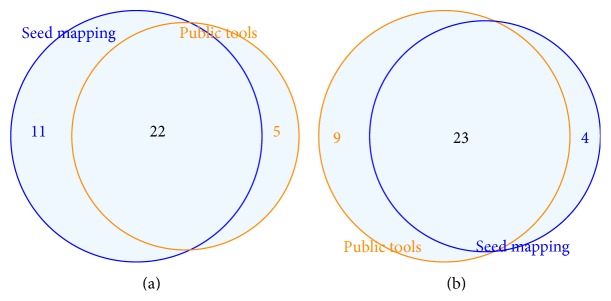
Venn diagrams showing overlap between nodes in coexpression networks ((a) miR-null network, (b) miR-142 network), seed mapping, and targets predicted by public tools like TargetScan, DIANA-microT, and miRanda. A R package [33] was used to create these figures.

While comparing the nodes in the enriched networks, their seed mapping, and other miRNA target prediction tools such as TargetScan [19], DIANA-microT [25], and miRanda [26], we observed that a large proportion of the seed mapping nodes in the networks overlap with the targets predicted from those publicly available tools (see Figure 5). The Chi-square test was inconclusive to suggest any relation between occurrence in coexpressed networks and preference to either of the seed mapping/publicly available methods (*χ* ^2^ = 4.38, df = 2, and *P* value = 0.1119).

### 3.4. Functional Analysis Reveals DE Genes Enriched in Nervous System

The individual networks (miR-null and miR-142) only contained around 50 nodes (genes). With this list of genes, no significant functional enrichment was observed. Next, we considered differentially expressed genes with adjusted *P* value ≤ 0.1. This list consisted of 85 genes and the functional enrichment for this list is shown in Table 5. Among several GO terms enriched, the table shows that all these terms are related to neuron and nervous system.

## 4. Discussion and Conclusion

Network analysis in gene expression profiling study is one of the rising trends in bioinformatics. Although the correlation does not imply causation, in a microRNA overexpression experiment, we have shown that a network model can be applied to extract meaningful biological relationships. The combined use of microRNA-mRNA 3′ UTR seed mapping and differential gene expression can further strengthen the efficacy of network analysis.

**Figure 6:**
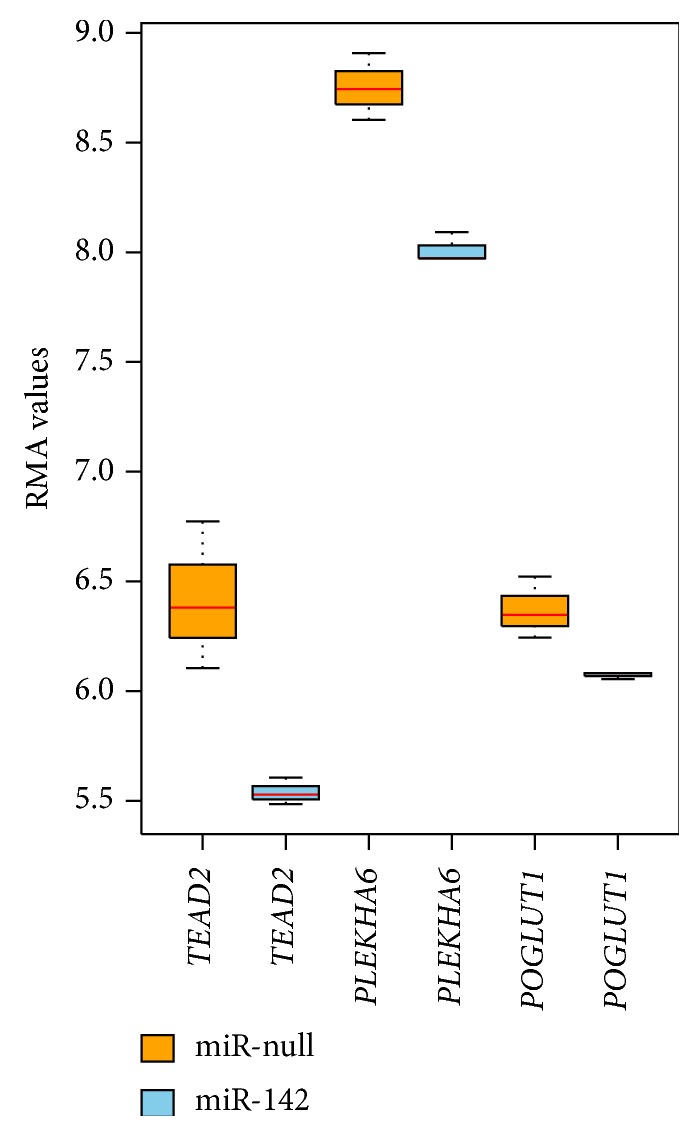
Expression values of* TEAD2*,* PLEKHA6*, and* POGLUT1* genes in miR-null and miR-142 samples. The first box for each gene represents gene expression in miR-null sample and the second box represents the same for miR-142 overexpressed sample.* TEAD2* and* PLEKHA6* were found differentially expressed using both the “Limma” package and “WRS” test (*P* value ≤ 0.1), while* POGLUT1* was found differentially expressed in the “WRS” *t*-test (*P* value ≤ 0.1).

By comparing the enriched networks in different experimental conditions, we showed that several genes were greatly affected by the respective treatment, namely, miRNA-142 overexpression. The results obtained from the “Limma” package show that the genes* TEAD2* and* PLEKHA6*, which appear in miR-142 overexpression network and have seed sequence mapping, are downregulated. Similar results obtained from Wilcoxon Rank Sum test show* POGLUT1* in addition to* TEAD2* and* PLEKHA6*.  Figure 6 shows the downregulation effect on these genes due to the overexpression of miR-142. The functional enrichment of differentially expressed genes using “Limma” package shows the enrichment of neuron and nervous system related GO terms. Next, we discuss the relevance of these genes in neuronal cells.

### 4.1. *TEAD2* Gene and Nervous System Development

The* TEAD2* gene encodes for* Tead2* transcription factor, which is one of the first transcription factors expressed at the beginning of mammalian development [27]. In 2007, [28] showed that this gene is required during neural development specifically for the neural tube closure. Recently, in 2014, [29] showed that* Tead2* together with* Yap* and* Taz* controls the expression of genes critical for epithelial-mesenchymal transition (EMT). EMT has long been known as an essential process for neural tube formation. In our study, we observed that this gene is differently coexpressed in miR-142 overexpressed network, downregulated in miR-142 overexpression, and has exact seed mapping for miRNA-142-5p. These observations and its role in nervous system development further demonstrate that it is a crucial target in miR-142 overexpression.

### 4.2. *PLEKHA6* Gene and Schizophrenia

*PLEKHA6* gene encodes for* pleckstrin homology (PH) domain containing family A member 6* protein. Recently, in 2014, [30] found that* PLEKHA6* is involved in intracellular signaling and associated its polymorphisms with schizophrenia. The authors suggested that this gene might be involved in the pathophysiology of schizophrenia and the therapy response towards antipsychotics. In our study, we found that* PLEKHA6* was present in our miR-142 network and was downregulated in miR-142 overexpression sample. It also contained inexact mapping of both miR-142-5p and miR-142-3p seed sequences in the 3p UTR. Studies have shown that this seed sequence mapping is not perfect. Allowing a mismatch in miRNA seed sequence can increase the regulation of diverse targets leading to the coexpression of these target genes. The correlation network captures this information and can serve as a better model to extract such information from these studies.

### 4.3. *POGLUT1* Gene and Notch Signaling Pathway

*POGLUT1* (also known as* RUMI* or* hCLP46*) is a gene that encodes for protein O-glucosyltransferase 1. It is shown that this gene is required for Notch signaling [31]. One of the major functions of Notch signaling is the neuronal function and its development. In [32], the authors have showed that* hCLP46* is the homolog of* Rumi* and its knockdown may result in Notch signaling impairment. Like* TEAD2*, we also found that* POGLUT1* was differently coexpressed in miR-142 network, was downregulated by miR-142 overexpression, and contained miR-142-5p exact seed mapping in its 3p UTR. These observations suggest that* POGLUT1* is another significant target in miR-142 overexpression.

In this study, we have identified candidate genes such as* TEAD2*,* PLEKHA6*, and* POGLUT1* that were highlighted in our enriched network and were greatly impacted by miRNA-142 overexpression. Most importantly, these genes were known to have very crucial neuronal functions.

## Figures and Tables

**Table 1:** Seed sequence of miR-142 and all possible one-base mutation. Bold base indicates mutated site.

Original/mutated	Seed sequence	
	miR-142-5p	miR-142-3p
Original	AUAAAGU	GUAGUGU
hsa-miR-142-1a	**U**UAAAGU	**A**UAGUGU
hsa-miR-142-1b	**G**UAAAGU	**U**UAGUGU
hsa-miR-142-1c	**C**UAAAGU	**C**UAGUGU
hsa-miR-142-2a	A**A**AAAGU	G**A**AGUGU
hsa-miR-142-2b	A**G**AAAGU	G**G**AGUGU
hsa-miR-142-2c	A**C**AAAGU	G**C**AGUGU
hsa-miR-142-3a	AU**U**AAGU	GU**G**GUGU
hsa-miR-142-3b	AU**G**AAGU	GU**U**GUGU
hsa-miR-142-3c	AU**C**AAGU	GU**C**GUGU
hsa-miR-142-4a	AUA**U**AGU	GUA**A**UGU
hsa-miR-142-4b	AUA**G**AGU	GUA**U**UGU
hsa-miR-142-4c	AUA**C**AGU	GUA**C**UGU
hsa-miR-142-5a	AUAA**U**GU	GUAG**A**GU
hsa-miR-142-5b	AUAA**G**GU	GUAG**G**GU
hsa-miR-142-5c	AUAA**C**GU	GUAG**C**GU
hsa-miR-142-6a	AUAAA**A**U	GUAGU**A**U
hsa-miR-142-6b	AUAAA**U**U	GUAGU**U**U
hsa-miR-142-6c	AUAAA**C**U	GUAGU**C**U
hsa-miR-142-7a	AUAAAG**A**	GUAGUG**A**
hsa-miR-142-7b	AUAAAG**G**	GUAGUG**G**
hsa-miR-142-7c	AUAAAG**C**	GUAGUG**C**

**Table 2:** Overlap between differentially expressed (DE) genes and miRNA seed targets. The first three rows represent exact seed sequence mapping and the last three represent inexact seed sequence mapping. “*P*” represents adjusted *P* values for the differential analysis. There are genes, which are not mapped to any miR-142 seed sequence but are not shown in this table.

Common genes	DE (*P* ≤ 0.1)	
	Down	Up
miR-142-5p	12	12
miR-142-3p	3	1
miR-142-5p/3p (both)	2	1

Inexact miR-142-5p	9	2
Inexact miR-142-3p	5	1
Inexact both	12	3

Total number of genes	60	25

**Table 3:** Size of miR-142 and miR-null networks.

	Number of nodes	Number of edges	Edge density
miR-null	57	217	0.136
miR-142	52	158	0.119

**Table 4:** Count of nodes/genes, which have seed sequence mapping in human genome and are present in networks.

	miR-null	miR-142	Genome
miR-142-5p	9	6	2914
miR-142-3p	1	2	720
miR-142-5p/3p (both)	—	—	387

Inexact miR-142-5p	7	7	3644
Inexact miR-142-3p	7	8	2520
Inexact miR-142-5p/3p (both)	9	4	4820

**Table 5:** Results from GO functional analysis of differentially expressed genes.

GO ID	Name	Total	In DE list	*P* value	Adjusted *P* value
GO:0042995	Cell projection	1451	22	2.62495317149281*e* − 07	0.00313028071281764
GO:0097458	Neuron part	903	16	2.19851448246544*e* − 06	0.0131087433407394
GO:0043005	Neuron projection	726	14	3.97485763192762*e* − 06	0.0144904323637896
GO:0030425	Dendrite	360	10	4.86048503367531*e* − 06	0.0144904323637896
